# Inferring yeast cell cycle regulators and interactions using transcription factor activities

**DOI:** 10.1186/1471-2164-6-90

**Published:** 2005-06-10

**Authors:** Young-Lyeol Yang, Jason Suen, Mark P Brynildsen, Simon J Galbraith, James C Liao

**Affiliations:** 1 Department of Chemical Engineering, University of California, Los Angeles, California, 90095, USA

## Abstract

**Background:**

Since transcription factors are often regulated at the post-transcriptional level, their activities, rather than expression levels may provide valuable information for investigating functions and their interactions. The recently developed Network Component Analysis (NCA) and its generalized form (gNCA) provide a robust framework for deducing the transcription factor activities (TFAs) from various types of DNA microarray data and transcription factor-gene connectivity. The goal of this work is to demonstrate the utility of TFAs in inferring transcription factor functions and interactions in *Saccharomyces cerevisiae *cell cycle regulation.

**Results:**

Using gNCA, we determined 74 TFAs from both wild type and *fkh1 fkh2 *deletion mutant microarray data encompassing 1529 ORFs. We hypothesized that transcription factors participating in the cell cycle regulation exhibit cyclic activity profiles. This hypothesis was supported by the TFA profiles of known cell cycle factors and was used as a basis to uncover other potential cell cycle factors. By combining the results from both cluster analysis and periodicity analysis, we recovered nearly 90% of the known cell cycle regulators, and identified 5 putative cell cycle-related transcription factors (Dal81, Hap2, Hir2, Mss11, and Rlm1). In addition, by analyzing expression data from transcription factor knockout strains, we determined 3 verified (Ace2, Ndd1, and Swi5) and 4 putative interaction partners (Cha4, Hap2, Fhl1, and Rts2) of the forkhead transcription factors. Sensitivity of TFAs to connectivity errors was determined to provide confidence level of these predictions.

**Conclusion:**

By subjecting TFA profiles to analyses based upon physiological signatures we were able to identify cell cycle related transcription factors consistent with current literature, transcription factors with potential cell cycle dependent roles, and interactions between transcription factors.

## Background

Transcription factor activities (TFAs) rather than levels of transcription factor expression mediate transcriptional regulations. Various post-transcriptional and post-translational modifications abolish significant correlations between TFAs and the level of transcription factor expression. Therefore, a strategy to determine the physiological functions and interactions of transcription factors based on the analysis of TFA profiles would be more fundamentally sound than one based on transcript levels *per se*. However, owing to post-translational modifications, TFAs are difficult to measure experimentally and therefore, many analyses infer TFAs by computational analysis of target gene expression levels either from single regulatory factors alone or in combination [1-10]. Among these, Network Component Analysis (NCA) [3] and generalized NCA (gNCA) [2] provide a robust framework for deducing TFAs based on DNA microarray data, promoter connectivity, and genetic regulatory constraints imposed by regulatory knock-out experiments. Using TFA profiles deduced by NCA and gNCA, we are now in a position to assign transcription factor functions and reconstruct functional interactions between transcription factors.

The goal of this work is to demonstrate how we analyze TFA profiles to determine physiologically relevant characteristics of transcription factors. Specifically, by analyzing TFA profiles of the *S. cerevisiae *cell cycle we identify cell cycle regulators and putative interaction partners of two forkhead transcription factors (Fkh1 and Fkh2) that are responsible for expression of a gene cluster within the M/G1 interval and involved in mitotic exit [11].

Once TFA profiles are determined, the transcription factor regulators and interactions are deduced based on the following hypotheses: 1) Transcription factors with similar activity patterns function together; 2) Transcription factors involved in cell cycle regulation exhibit oscillatory activity patterns, 3) Activities of transcription factors which functionally interact with each other will be disturbed if one of the interacting partners is deleted. These hypotheses are in spirit similar to the traditional analysis of gene expression. In contrast to traditional analysis of gene expression, our analyses are constructed and applied directly to TFA profiles.

## Results

### Overall strategy

**Figure 1:**
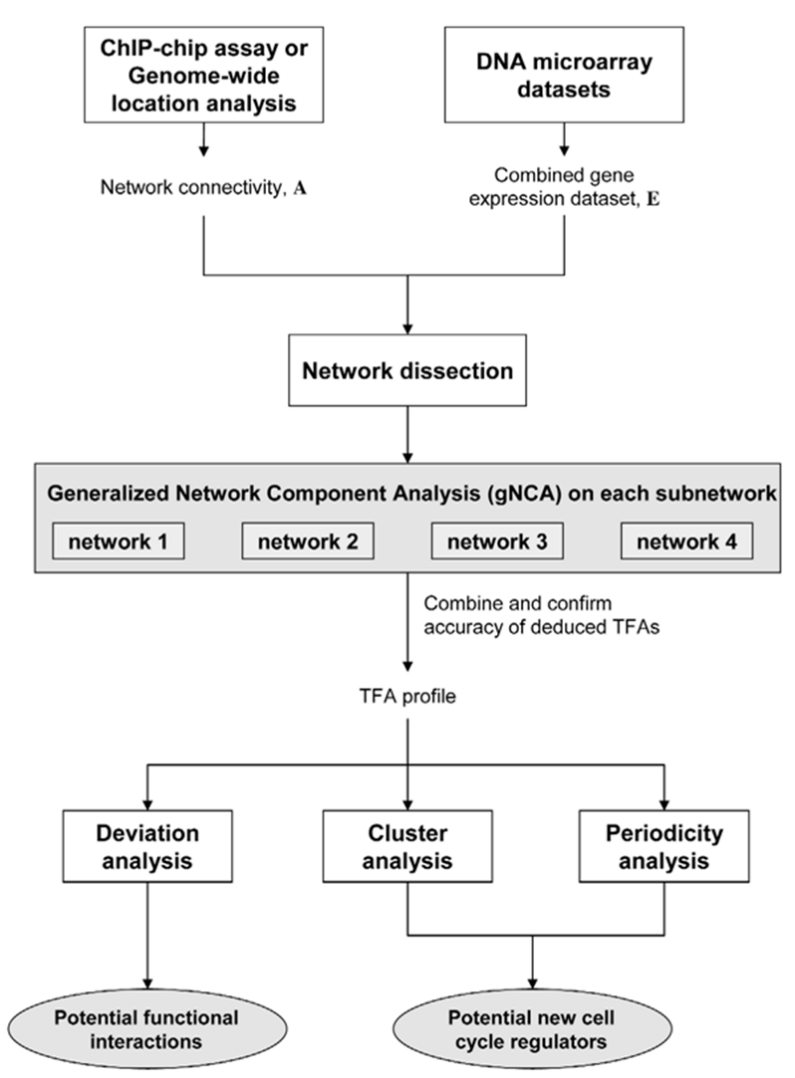
**Flowchart summarizing our methodology**. Flowchart of the method used to determine transcription factor functions and interactions.

A flow diagram demonstrating the relationships between each component of the methodology is presented in Figure 1. Given the gene expression data and connectivity network between transcription factors and gene expression, we first deduced TFAs from gene expression by using gNCA. The deduced TFAs were then analyzed by two complementary methods. First, we used cluster analysis [12] to group TFAs that behave in a similar manner. Second, a statistical analysis [4] was used to determine if the signal from each of the TFAs was periodic. The results from these two analyses were integrated to deduce putative cell cycle regulators. We hypothesized that putative cell cycle regulators should exhibit periodic activity profiles and cluster closely with known cell cycle regulators. Then TFA profiles from wild type and the *fkh1 fkh2 *mutant strain under the same experimental conditions were statistically analyzed to deduce transcription factors whose profiles were significantly perturbed, thus identifying putative functional interaction partners of the fork-head transcription factors.

### Application of gNCA to the combined wild-type and mutant data set

We used gNCA to analyze both the wild-type [13] and the *fkh1 fkh2 *mutant data [11]. The wild-type data and the TF-knockout data were organized in different columns of the **E **matrix (expression data matrix where *E*_*ij *_corresponds to the log of gene expression ratio of gene *i *evaluated at experiment *j*, see Methods). The elements in **P **(TFA matrix where *P*_*ij *_corresponds to the log of relative transcription factor activity of TF *i *at experiment *j*, see Methods) corresponding to the specific knockout strain were kept at zero, in addition to the zero constraints placed in **A **(control strength matrix where *A*_*ij *_denotes the control strength of transcription factor *j *on gene *i*, see Methods) according to the transcription factor-gene connectivity information.

**Figure 2:**
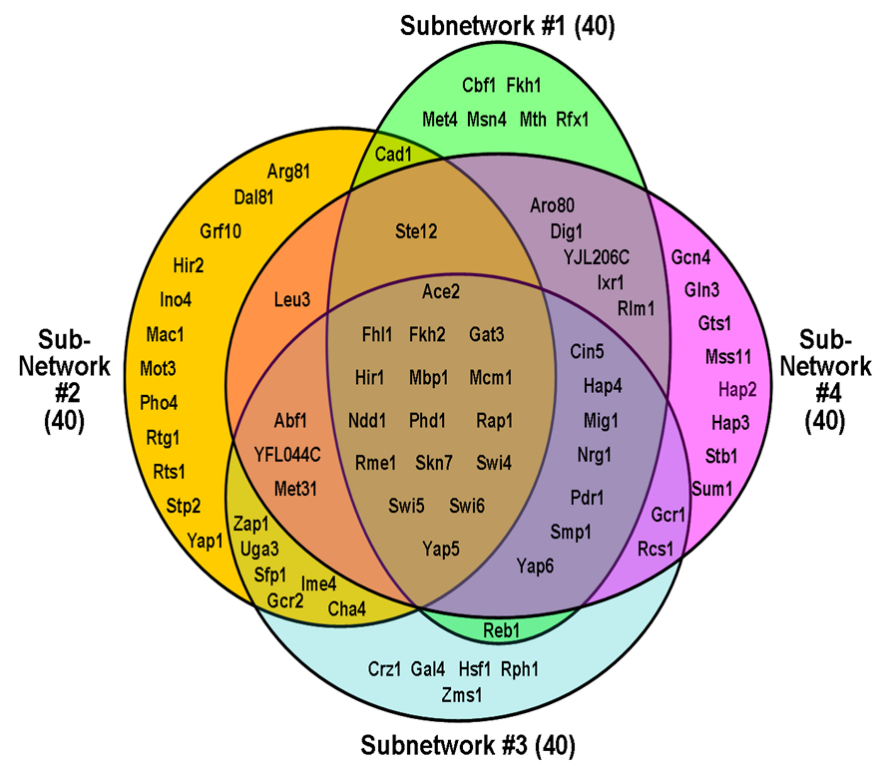
**Venn diagram of overlapped transcription factors among the four sub-networks analyzed**. Each sub-network contains 40 transcription factors, but together, 74 Transcription factors can be used for determining TFAs. Sub-network 1, 2, 3, and 4 contains 1110, 847, 1015, and 793 genes, respectively. Total 1529 genes were finally selected for generating 4 multiple sub-networks from 1818 genes with full data points in the combined dataset.

The first step in gNCA was to select sub-networks based on available transcription factor-gene connectivity [14]. Each sub-network was constructed to satisfy the gNCA criteria. gNCA requires that the number of transcription factors (*L*) in each sub-network be less than that of data points (*M*) in the data matrix. In the combined cell cycle microarray data set, *M *= 69, and thus the number of transcription factor in gNCA in each analysis cannot exceed 69. Thus, subsets of the 104 transcription factors included in the genome-wide location analysis [14] were selected to form sub-networks for gNCA. Overlapping random sub-networks were generated and screened using the gNCA criteria. Among them, four that satisfy the gNCA criteria were selected in order to show that using multiple sub-networks makes it possible to determine TFAs more than the number of data points (M = 69). Each sub-network contained 40 transcription factors, but together, a total of 74 transcription factors were analyzed. The transcription factors involved in each of the 4 sub-networks are shown in Figure 2. Only 16 transcription factors among 74 transcription factors were fully overlapped among all 4 sub-networks.

**Figure 3:**
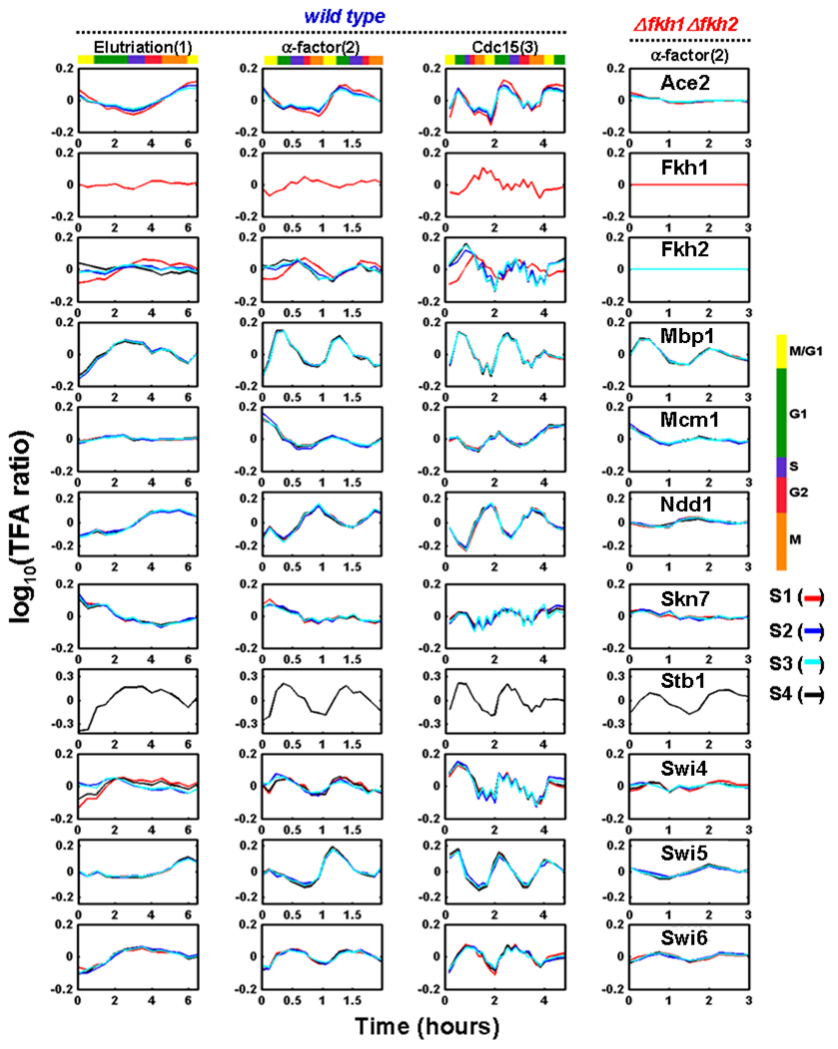
**Comparison of TFA profiles of 11 major yeast cell cycle related transcription factors between wild type and *fkh1 fkh2 *mutant**. The first 3 columns corresponds to data deduced from yeast cultures synchronized by elutriation, α-factor arrest, and arrest of a *cdc15 *temperature-sensitive mutant which can give one cell cycle, two cell cycles, and three cell cycles for given experimental measurements, respectively. The last column corresponds to *fkh1 fkh2 *double knock-out mutant synchronized with α-factor arrest. Different stages in the cell cycle are indicated by the color code. Different colors in TFA profiles for each transcription factor represent TFAs from different sub-networks. S1, S2, S3 and S4 represent sub-networks 1, 2, 3, and 4, respectively.

After applying gNCA independently to the 4 sub-networks, 74 TFAs were determined. Figure 3 shows the TFAs of the 11 known cell cycle factors from 4 different sub-networks. Qualitatively, these TFAs appear to show expected oscillatory behavior for 1, 2, and 3 cycles according to the method of synchronization (elutriation, α-factor arrest, and *cdc15 *mutation). The dynamics of overlapping TFA profiles between the four different sub-networks are very similar. This indicates that the deduced TFAs were insensitive to the sub-network selection. In addition, combining the *fkh1 fkh2 *mutant data with the wild-type data yields similar TFAs predicted from wild-type data alone [3], suggesting the consistency between the two data sets.

It is recognized that most transcription factors are regulated in post-transcriptional processes. Thus, their activity may deviate significantly from the expression level as noted previously [3]. For 27 transcription factors with both gene expression level and TFA available, the correlation coefficients ranged from - 0.5 to 0.6. The wide range difference in the correlation coefficients confirms that TFAs and expression levels exhibit very different dynamic profiles and therefore TFAs cannot be substituted by expression levels in analysis.

### Sensitivity Analysis

To assess the effect of connectivity errors on our results, we altered up to 10% of the connectivity graph for every subnetwork, by randomly deleting and inserting connections between transcription factors and genes. This effectively simulates the presence of false negatives and false positives in ChIP-chip assays. Subsequently, we ran gNCA on our randomly perturbed graphs and compared the deduced TFAs to our reported results via the correlation coefficient. The procedure was performed 100 times and the TFAs deduced from each run were compared with the TFAs computed with unaltered connectivity using Pearson correlation coefficients (Table 1). Transcription factors with high correlation coefficients suggest robustness of the results against connectivity errors.

**Table 1:** Sensitivity of TFA profiles to errors in connectivity from ChIP-chip assay. Connectivity errors were simulated by randomly inserting and deleting connections in the connectivity graph generated from the ChIP-chip assay. gNCA was performed on the perturbed connectivity, and the resultant TFAs were compared to those from un-perturbed connectivity via the correlation coefficient. This procedure was performed on each of the 4 subnetworks 100 times. The average correlation coefficients for transcription factors in the 4 subnetworks are presented here. A low correlation coefficient (<0.5) suggests sensitivity to network connectivity error.

**Subnetwork 1**	**Mean Correlation Coefficient**	**Subnetwork 2**	**Mean Correlation Coefficient**	**Subnetwork 3**	**Mean Correlation Coefficient**	**Subnetwork 4**	**Mean Correlation Coefficient**
ABF1	0.941	ABF1	0.951	ABF1	0.94	ACE2	0.912
ACE2	0.953	ACE2	0.857	ACE2	0.897	ARO80	0.657
ARO80	0.645	ARG81	0.286	CHA4	0.362	CIN5	0.601
CAD1	0.511	CAD1	0.659	CIN5	0.59	DIG1	0.718
CBF1	0.401	CHA4	0.448	CRZ1	0.313	FHL1	0.992
CIN5	0.651	DAL81	0.581	FHL1	0.993	FKH2	0.669
DIG1	0.686	FHL1	0.99	FKH2	0.657	GAT3	0.493
FHL1	0.992	FKH2	0.635	GAL4	0.492	GCN4	0.601
FKH1	0.614	GAT3	0.457	GAT3	0.511	GCR1	0.321
FKH2	0.535	GCR2	0.425	GCR1	0.306	GLN3	0.402
GAT3	0.446	GRF10(Pho2)	0.565	GCR2	0.446	GTS1	0.227
HAP4	0.706	HIR1	0.732	HAP4	0.769	HAP2	0.375
HIR1	0.472	HIR2	0.357	HIR1	0.465	HAP3	0.47
IXR1	0.511	IME4	0.357	HSF1	0.981	HAP4	0.856
MBP1	0.946	INO4	0.419	IME4	0.442	HIR1	0.32
MCM1	0.558	LEU3	0.575	MBP1	0.979	IXR1	0.475
MET31	0.436	MAC1	0.723	MCM1	0.572	LEU3	0.518
MET4	0.381	MBP1	0.985	MET31	0.435	MBP1	0.951
MIG1	0.336	MCM1	0.701	MIG1	0.354	MCM1	0.705
MSN4	0.444	MET31	0.507	NDD1	0.965	MIG1	0.33
MTH1	0.463	MOT3	0.413	NRG1	0.421	MSS11	0.404
NDD1	0.958	NDD1	0.971	PDR1	0.366	NDD1	0.962
NRG1	0.495	PHD1	0.541	PHD1	0.583	NRG1	0.563
PDR1	0.334	PHO4	0.599	RAP1	0.917	PDR1	0.372
PHD1	0.569	RAP1	0.856	RCS1	0.48	PHD1	0.64
RAP1	0.917	RME1	0.407	REB1	0.576	RAP1	0.897
REB1	0.643	RTG1	0.413	RME1	0.415	RCS1	0.424
RFX1	0.493	RTS2	0.302	RPH1	0.294	RLM1	0.553
RLM1	0.519	SFP1	0.564	SFP1	0.582	RME1	0.389
RME1	0.367	SKN7	0.536	SKN7	0.669	SKN7	0.609
SKN7	0.545	STE12	0.518	SMP1	0.619	SMP1	0.642
SMP1	0.614	STP2	0.322	SWI4	0.553	STB1	0.576
STE12	0.79	SWI4	0.592	SWI5	0.987	STE12	0.738
SWI4	0.747	SWI5	0.963	SWI6	0.61	SUM1	0.823
SWI5	0.991	SWI6	0.648	UGA3	0.392	SWI4	0.843
SWI6	0.606	UGA3	0.383	YAP5	0.512	SWI5	0.991
YAP5	0.863	YAP1	0.565	YAP6	0.404	SWI6	0.589
YAP6	0.56	YAP5	0.657	YFL044C	0.578	YAP5	0.572
YFL044C	0.583	YFL044C	0.546	ZAP1	0.442	YAP6	0.595
YJL206C	0.396	ZAP1	0.527	ZMS1	0.295	YJL206C	0.455

### Determination of cell cycle-dependent regulators based on TFA dynamics

**Figure 4:**
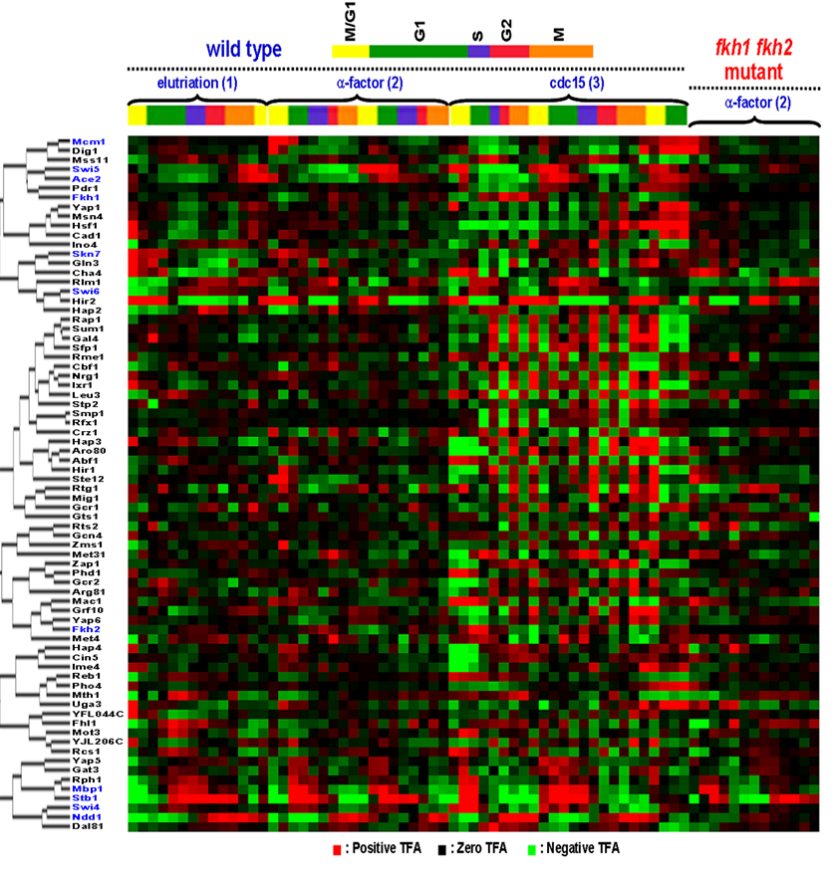
**Hierarchical clustering of TFAs of all 74 Transcription factors**. Absolute correlation coefficient as a similarity measure and the average linkage method were used for clustering. Green, red, and black color represents negative log TFA ratios, positive log TFA ratios, and 0, respectively. The color intensity increases as the magnitude of each TFA value increases. The TFs denoted in blue are those TFs known to be involved in cell cycle.

We hypothesized that transcription factors with similar activity patterns are involved in related processes. Thus, clustering of TFAs would allow identification of transcription factors related to cell cycle regulation. After hierarchical clustering (Figure 4), 11 transcriptional factors (Dal81, Dig1, Gat3, Hap2, Hir2, Mss11, Pdr1, Rlm1, Rph1, Yap5, and Yap6) were found to cluster closely with the 11 known cell cycle regulators.

**Figure 5:**
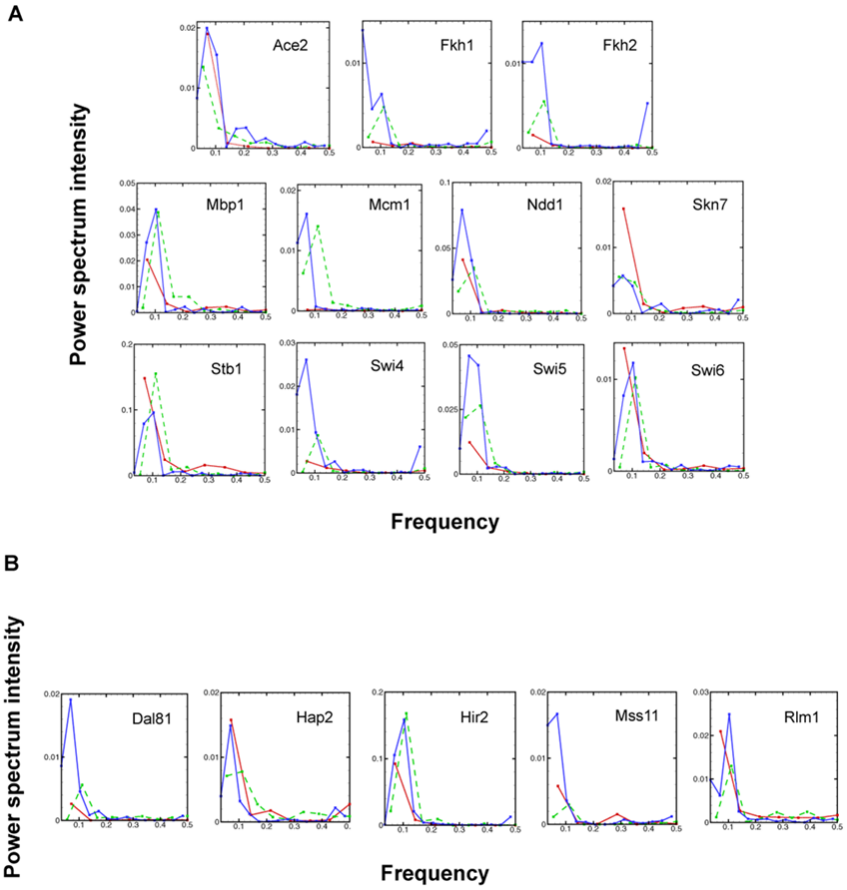
**Power spectra for selected TFAs from the 4 different sub-networks**. (A) 11 known cell regulators. (B) The top 5 TFAs that exhibits periodic function. In both sub-figures, solid blue lines are the data deduced from yeast cultures synchronized by arrest of a *cdc15 *temperature-sensitive mutant, dash green lines are data deduced from yeast cultures synchronized by α-factor arrest, and dotted red lines are data collected from yeast cultures synchronized by elutriation.

In addition to cluster analysis, we performed a statistical test to identify the set of TFAs with a periodic profile. Figure 5A shows the power spectra of the 11 known cell cycle regulator. A power spectrum is a representation of a signal in the frequency domain. A dominant peak in the power spectrum corresponds to the fact that the underlying process has a principal oscillation frequency. Most of the power spectra to identify periodic patterns exhibit a single strong peak at low frequency. This suggests that the dynamic profiles appear to be periodic. To classify whether a signal is periodic or not, we employed a statistical criterion [4] of rejecting the null hypothesis of purely random process. The result suggests that 9 out of 11 known cell cycle regulators exhibit periodic behavior. This confirms that the activity profiles of cell cycle regulators are periodic. We then applied the same periodicity test to the rest of 63 transcription factors and found that about 16 of the deduced TFAs from elutriation and α-factor arrest were statistically periodic and 44 of the deduced TFAs from cdc15 were statistically periodic. Combining the results of the periodicity test and cluster analysis, we found that 5 (Dal81, Hap2, Hir2, Mss11, and Rlm1) out of the 11 transcription factors closely clustered with the known cell cycle factors were statistically periodic and were regarded as putative cell cycle-related regulators.

**Figure 6:**
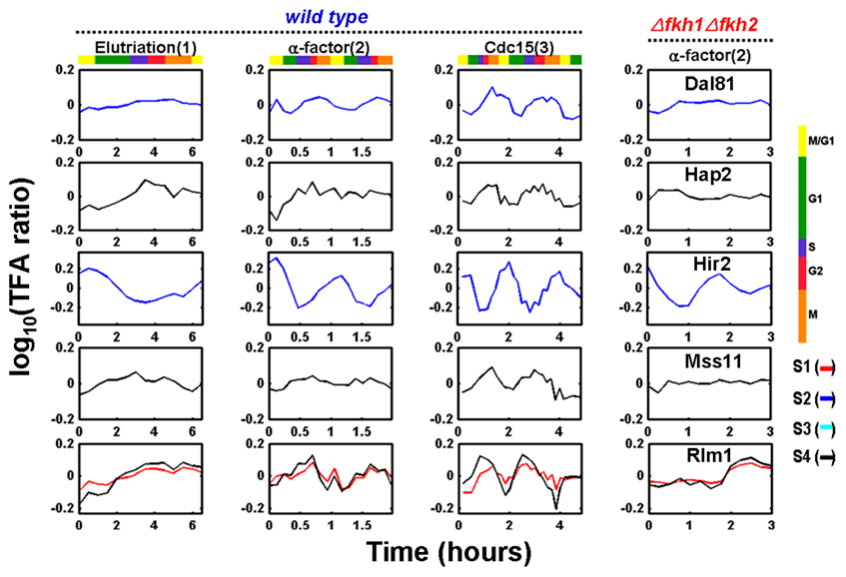
**TFA profiles of 5 putative cell cycle regulators**. Details of the figure legends are the same as Figure 3.

**Figure 8:**
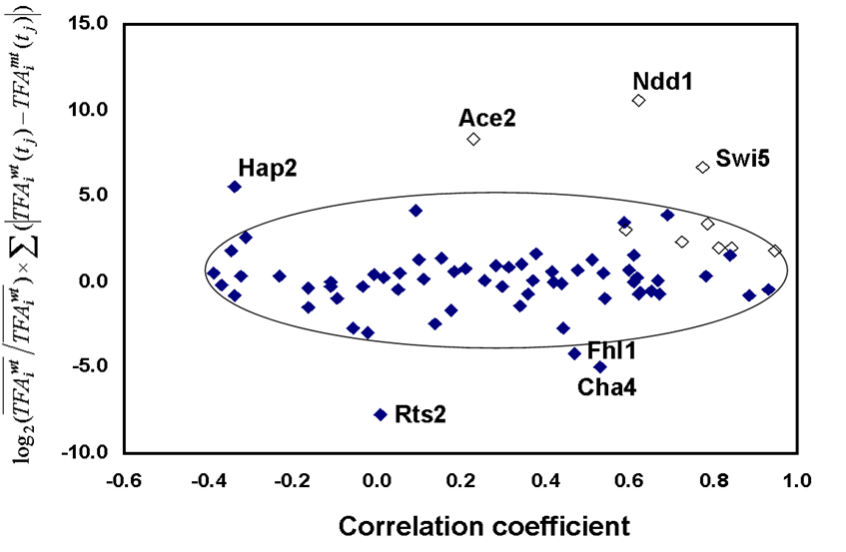
**Pearson correlation coefficients and the deviation coefficient for 11 known cell cycle factors (empty symbols) and other remaining 63 TFs (solid symbols) under the release from α-factor arrest**. The oval encloses TFAs with both low deviation coefficient.

The power spectra of these 5 putative cell cycle regulators are shown in Figure 5B, and the TFA profiles of these regulators are illustrated in Figure 6. The clustered TFA pattern of all cell cycle-related transcription factors (Figure 8) shows that the peak activities of these transcription factors gradually change from one phase to another through the cell cycle. Among the 5 putative cell cycle-related regulators, Hir2 is a regulator of histones [1,15], and it is therefore reasonable to expect it to have cell cycle related functions. It is not clear how the other 4 transcription factors are related to cell cycle regulation. Lee *et al*. [14] categorized Dal81 and Mss11 as metabolism regulators, Hir2 as a DNA/RNA/Protein biosynthesis regulator, and Rlm1 as environmental response regulators. These results suggested that transcription factors related to many other cellular processes may be involved in or dependent on cell cycle regulation to coordinate cellular processes [14].

### Functional interaction of FKH1 FKH2 with other transcription factors

Comparing the TFAs derived from the wild-type strain and the *fkh1 fkh2 *mutant under the same experimental conditions allows the determination of functional interactions between Fkh1 Fkh2 with other transcription factors. If such interaction exists, the activities of the interaction partners will change in the *fkh1 fkh2 *mutant. In general the *fkh1 fkh2 *mutant showed reduced oscillation amplitude as compared to the wild type (Figure 3). In particular, Ace2, Swi5, and Ndd1 showed significantly reduced oscillation in the *fkh1 fkh2 *mutant, suggesting potential interactions between these transcription factors and the two fork-head transcription factors. Indeed, it was found that Fkh2 forms a complex with Mcm1 and Ndd1 [16,17] which controls G2/M genes and also binds the promoters of Ace2 and Swi5 to activate genes at the following M/G1 phase. The reduced amplitude in TFAs of Ace2 and Swi5 in the *fkh1 fkh2 *mutant support that there is a cascade interaction between the two forkhead transcription factors and Ace2 and Swi5 [16].

TFA profiles of the wild-type strain (Figure 3) show that Ace2 and Swi5 were most active around M/G1 phase while Fkh1, Fkh2, Mcm1, and Ndd1 were most active at G2/M, G2, M/G1, and G2/M phase, respectively. These results are well consistent with previous results that Mcm1, Ace2, and Swi5 are M/G1 transcriptional regulators while the complex of Mcm1, Fkh2 and Ndd1 is a G2/M activator. Therefore, this result shows that TFA in a regulator knockout strain can be used to determine functional interactions.

By utilizing the Pearson correlation coefficient and deviation coefficient defined in the Methods section, Figure 8 shows that most transcription factors were aggregated in one cluster. Outside of this cluster are transcription factors which potentially were affected by the *fkh1 fkh2 *mutation and thus are functional interaction partners of the forkhead transcription factors. These interaction partners include Ace2, Ndd1, and Swi5, which are known to interact with Fkh2, and Hap2, Rts2, Cha4, and Fhl1, which were not known to interact with the forkhead transcription factors. In particular, Hap2 was also identified in the above analysis as a putative cell cycle-related regulator, supporting their functional interaction with the forkhead transcription factors. Cha4 and Hap2 were classified as metabolism-related factors, Fhl1 DNA/RNA/Protein synthesis, and Rts2 cell cycle and data processing [14]. Their modes of interaction with the forkhead transcription factors remain unknown.

## Discussion

Transcriptional regulators are commonly modified at the post-transcriptional level, and consequently their biological activities do not correlate significantly with expression levels. Previous work infer TFAs from expression levels of genes regulated by single factors or in combination [2,5-8,10]. In general, the major difference between previous work and that of NCA is that the former require an explicit quantification of the control strengths (the A matrix in NCA) *a priori*. Bussemaker et al. [7] defined the control strength as the motif copy number in corresponding promoters and found that there was no statistical benefit to model expression with more than single factors and thus deduced single TFAs. Wang et al. [6] considered an expression-weighted motif to find potential target genes of single transcription factors. Gao et al. [18] used ChIP-chip log occupancy ratios as a surrogate for transcription factor binding affinity. In contrast, NCA explicitly models combinatorial regulation of gene expression, and allows both the control strengths and the TFAs to be deduced simultaneously with given network connectivity. In this approach, the lack of connectivity in specific pairs of transcription factor and promoter is used to provide constraints for data decomposition in order to obtain unique solutions when specific criteria are satisfied [3]. gNCA expands these capabilities by allowing incorporation of constraints onto the deduced TFAs, such as transcription factor knockout experiments, which offer a rich source of data and biochemical information. Development of these methodologies significantly expands the capabilities of transcriptional regulation analysis. With gNCA, we analyzed the combined wild-type and *fkh1 fkh2 *mutant data set and showed that gNCA can be used to identify TFAs which are consistent with cell physiology, transcription factors with potential cell cycle dependent roles, as well as interactions between transcription factors.

On the basis that transcription factors exhibiting similar activity patterns function together, we identified 11 transcription factors that clustered closely with the known cell cycle regulators. We performed a periodicity test to determine the TFA profiles that exhibit periodic behavior, and by combining the sets of transcription factors collected by these two methods, we identified 5 putative cell cycle-related regulators: Dal81, Hap2, Hir2, Mss11, and Rlm1. These transcription factors may participate in functions driven by cell cycles, or may regulate cell cycle directly or indirectly.

Our comparison between the wild-type TFAs and the mutant TFAs confirmed that the forkhead transcription factors interact with Ace2, Ndd1, and Swi5. This result is consistent with previous reports [16,17]. Using this approach, we identified 4 additional transcription factors that may functionally interact with Fkh1 Fkh2 directly or indirectly: Hap2, Rts2, Cha4, and Fhl1. Most of these transcription factors are not known to be related to cell cycle, suggesting that cell cycle regulation interacts with other physiological functions.

It is worth noting that our analysis can be sensitive to errors in the connectivity graph. Through a sensitivity analysis we determined that all the known cell cycle regulators and all the known forkhead interaction partners have TFAs that exhibit low sensitivity to the connectivity network when using 0.5 as a correlation coefficient threshold. These results suggest that our analysis is robust to errors in connectivity. With the same sensitivity criterion, 2 (Dal81 and Rlm1) of the 5 putative cell cycle-related regulators and 1 (Fhl1) of the 4 putative forkhead interaction partners were determined to be robust to connectivity errors. The lack of sensitivity to error increases confidence in these predictions.

**Figure 9:**
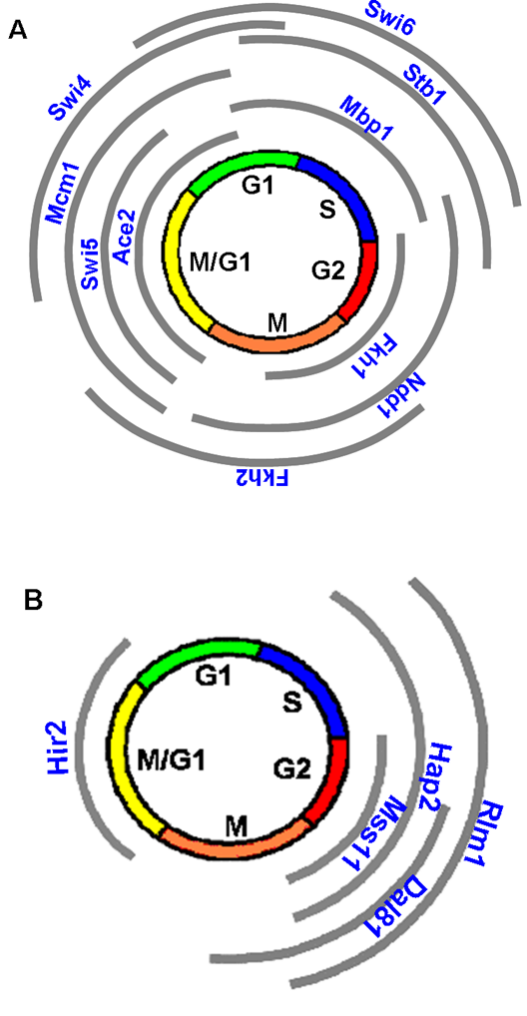
**Phase diagrams of TFAs**. (A) 11 known Transcription factors and (B) 5 deduced cell cycle-dependent factors.

A total of 1529 (out of 6200) genes and 74 (out of 104) transcription factors were analyzed from 69 microarray experiments. Both limited connectivity information from ChIP-chip and missing data points in DNA microarray expression attribute to the limited number of genes and TFs that can be studied in the current investigation. On the other hand, the results suggest that our analysis on this limited set of genes and TFs appears to be sufficient: our analysis strategy recovers nearly 90% of the known cell cycle regulators. On the basis of the TFA profiles, the time series of the key cell cycle regulators are summarized in Figure 9. A dominant feature in this map is the overlapping TFAs among known cell cycle regulators. This is common among some transcription factor complexes, including SBF (Swi4/Swi6) and MBF (Mbp1/Swi6), as well as transcription factors known to regulate the same phase in the cell cycle including Ace2, Swi5 and Mcm1 (Figure 9A). In addition, overlapping TFAs were also observed among different phases of cell cycle: transcription factors during one stage regulate transcription factors that function in the next stage. It is well known that serial regulation among transcription factors forms a connected regulatory network [17]. From the TFA profiles, we also observe the intrinsic property of the connected regulatory network among cell cycle factors in cell cycle regulation (Figure 9A).

**Figure 7:**
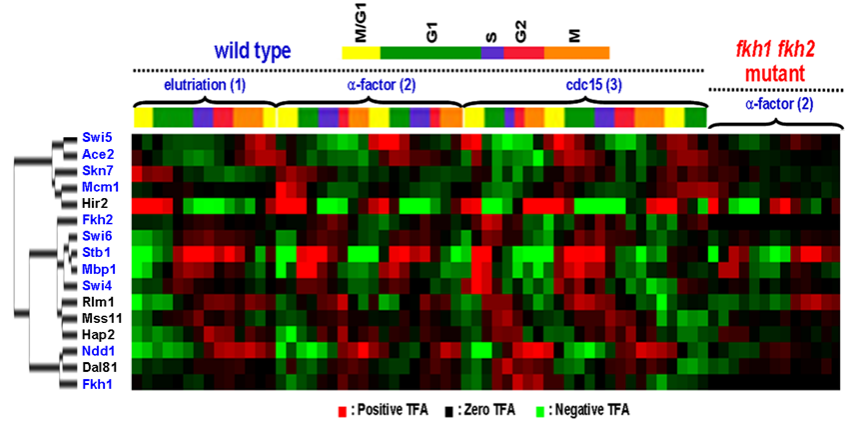
**Hierarchical clustering of TFA profiles of the 11 known cell cycle factors (blue) and the 5 putative cell cycle-dependent factors (black)**. TFs denoted in blue are those TFs known to be involved in cell cycle.

Among the putative cell cycle-related transcription factors (Figure 6), Dal81 is phosphorylated by Cdk1 [19] which is considered to be involved in G2/M transition [20]. The activity of Dal81 peaked over the G2/M phase is also in agreement with its regulatory role (Figure 9B). Hir2 functions as a transcriptional repressor of histone gene expression during the cell cycle [15]. Histone synthesis is triggered at the beginning of the S phase. So Hir2 is expected to have the lowest TFA around S phase. As such, TFA of Hir2 from NCA showed that it indeed has the lowest TFA around S phase (Figure 7). Most well characterized transcription factors showed biologically relevant activity profiles at specific cell cycle phases, suggesting that TFAs deduced from gNCA are biologically meaningful and a good predictor of transcription factor function and interaction.

## Conclusion

Protein function and interaction are often deduced computationally through analysis of genomic sequence, protein sequence, domain architecture, phylogenic profile, or gene expression level. Gene expression analysis represents the only current technique that takes into account dynamic behavior to assign interaction and function. However, it has been shown that for proteins significantly regulated post-transcriptionally (e.g. transcription factors) their gene expression levels do not correlate well with their activity. Here a method for screening the physiological roles of transcription factors and their functional interactions based on their dynamic activity profiles was introduced. Our method first determines TFAs, which were then further analyzed with cluster and periodicity analysis to determine transcription factor function. By combining the results from both cluster analysis and periodicity analysis, we recovered more than 90% of transcription factors that are proposed to be involved in cell cycle regulation. In addition, we discovered 5 putative transcription factors that may be related to cell cycle regulation. Functional interactions between transcription factors were determined by isolating statistically significant perturbations in TFA patterns between mutant Δ*fkh1*Δ*fkh2 *and wild-type experiments carried out under the same experimental conditions. This method allowed for the identification of 4 novel and 3 previously verified fork-head transcription factor interaction partners. We recognize that the Chip-chip data may be condition-dependent and noisy. However, together with microarray data, they provide useful information for functional deduction of transcription regulators.

## Methods

### Gene expression data and connectivity information

In this study, we utilized microarray data sets which were taken from wild-type *S. cerevisiae *cultures synchronized by three independent methods, α-factor arrest, elutriation, and arrest of a *cdc15 *temperature-sensitive mutant [13] and a mutant that lacks in two forkhead transcription factors [11], Fkh1 and Fkh2 synchronized by α-factor arrest. The two data sets were combined after imputation [21] to fill in missing points, and common genes without missing data points between the two were selected for NCA. The wild type and the mutant data were organized side-by-side in different columns of the data matrix. The total number of microarray experiments in the combined dataset was 69.

The connectivity information between transcription factors and their regulated genes comes from the genome-wide location or ChIP-chip assay [14]. In this study, the *p*-value threshold used was 0.001 for determining connectivity. As with any other method for determining genome wide transcriptional networks, the ChIP-chip assay suffers from miss-connectivity issues. Both under- and over-prediction errors in connectivity can be rationalized to occur in genome wide location analysis. Since NCA deductions are dependent on the connectivity graph generated by ChIP-chip assays, we endeavoured to determine the effects of connectivity errors on our TFA profiles.

### gNCA

NCA and its extension, gNCA, are a recent-developed technique that is capable of deducing TFAs based on gene expression. Compared to NCA, a distinct feature of gNCA is the capability of imposing constraints on both the regulatory network and regulatory signals. This considerable expands the range of network topologies capable of being analyzed with NCA, and thus allows application of the method to a much larger expanse of scenarios. The analysis starts with defining a model that expresses gene expressions as a function of TFA and the corresponding control strengths (CS). The resulting model can be written in a log-log form. In matrix notation, this model is:

**E = A × P + Γ**,     **(1)**

where *E*_*ij *_= log(*g*_*i*_(*t*_*j*_) / *g*_*i*_(*t*_0_)) and *g*_*i*_(*t*_*j*_) is the expression of *i*-th gene evaluated at time *t*_*j*_**, A **is the regulatory network of control strengths where *A*_*ij *_denotes the control strength of transcription factor *j *on gene *i *(*A*_*ij *_*= CS*_*ij*_), *P*_*ij *_= log(TFA_*i *_(*t*_*j*_) / TFA_*i *_(*t*_0_)) with TFA_*i *_(*t*_*j*_) being the *i*-th transcription factor activity evaluated at time *t*_*j*_, and **Γ **represents external stimulus and stochastic background noise from the DNA microarray. The dimensions of **E**, **A **and **P **are (*N *× *M*), (*N *× *L*) and (*L *× *M*), respectively, where *N *is the number of genes in the network, *M *is the number of data points or experiments conducted, and *L *is the number of transcription factors used in the study. Since most genes are regulated by only a subset of transcription factors, **A **is generally sparse. This sparse connectivity pattern in **A **is defined by *Z*_A_, which can be obtained from ChIP-chip assay or existing databases. In addition, gNCA can impose constraints on **P**. For example, when a gene that codes for a specific transcription factor is deleted, the TFA profile (represented in the P matrix) is set to zero. The corresponding zero pattern of **P **is denoted as *Z*_P_. If *Z*_A _and *Z*_P _satisfy a given set of conditions, then gNCA can decompose the data **E **into **A **and **P **up to a diagonal scaling factor. This condition is called *essential uniqueness *[3,22]. Additional technical details may be found elsewhere.

### Selection of multiple sub-networks for gNCA

Among the set of required conditions for an *essentially unique *decomposition, a necessary condition is that the number of transcription factors (*L*) must be less than the number of data points (*M*) in a given network. Therefore, to analyze a large network in yeast, we dissected the regulatory network into multiple sub-networks and recombine them at the end of the analysis. Each sub-network was constructed such that the number of transcription factors was less than the number of time data points.

Satisfaction of the NCA identifiablity criteria [3,22] for each sub-network requires that each sub-network contain some overlapping TFs to ensure *consistency *among the computed dynamics. This is possible because the nature of the transcriptional regulatory network as determined by genome-wide location analysis data reveals that some transcript factors highly overlap the set of genes on the microarray [14]. Including these transcript factors in each sub-network decomposition step achieves the required consistency. First we select randomly *k *transcript factors from the column of A as an initial sub-network (*S'*). If this sub-network fails a rank check we then remove the *l *TFs that cause the rank deficiency, and replace them with *l *new TFs that were not used to create *S'*. The top *l *TFs are chosen by ranking the TFs by the total number of genes they control in descending order. If this continues to fail greater than a specified number of iterations, then we change *k *(usually to a smaller number) and repeat the process. For large enough *k *(40 TFs) it is likely that the many genes overlap because of the *a priori *network structure. This is by no means the only way to construct sub-networks, nor is it the optimal way. We do not guarantee that this will work in all cases, but for our purposes it is sufficient to generate consistent TFAs.

Among the combined data set with imputed missing data points, a total of 1818 genes with connectivity information of 104 TFs from genome wide location analysis were used to generate random sub-networks. Sub-networks with 40 transcription factors from total 104 TFs were generated and examined for gNCAuniqueness conditions. Four gNCA-compliant sub-networks, with 1110, 847, 1015, and 795 genes respectively, were chosen such that total number of TFAs deduced from 4 random sub-networks could be greater than the number of data points (M = 69). For each sub-network containing Fkh1 or Fkh2, the corresponding TFAs in the **P **matrix corresponding to the deletion mutant were constrained to zero.

### Cluster analysis of TFA profiles

The identification of potential new cell cycle factors was conducted by using hierarchical clustering on the TFA profiles. Specifically, hierarchical clustering based on absolute value of Pearson correlation coefficient, which measures the strength and direction of a linear relationship between two variables, was applied to TFA profiles deduced by gNCA. We hypothesize that transcription factors with similar activities function together. Therefore, transcription factors which are clustered together with 11 known cell cycle factors could be considered as possible cell cycle-dependent transcription factors. Publicly available software packages, Cluster and Treeview [12] were used for this test.

### Periodicity analysis of TFA profiles

We identify periodic functions from stochastic background noise by employing a statistical analysis technique described previously [4]. This technique is based on rejecting the null hypothesis of a purely random process through considering the power spectrum of a time dependent signal. First, the technique computes the power spectrum of a given signal and evaluates the *g*-statistics expressed as the contribution of the power spectrum at a specific frequency to the total intensity of the power spectrum. Large values of *g*-statistics suggest that the underlying process is periodic. Then, the signals are screened for periodic motion using multiple testing under the criterion of false discovery rate (FDR). The R package GeneTS. [4] was used for this study.

### Statistical analysis for interaction determination

The effect of *fkh1 *and *fkh2 *mutations on TFAs is elucidated by examining the correlation between TFAs from the wild type and the mutant under the same experimental condition. If there is a significant difference in TFAs between the wild type and mutant, the correlation coefficient should be low. However, low correlation coefficients may be caused by low overall activities, since in those situations the TFAs are dominated by noise. Therefore, we need to define a new deviation coefficient of TFAs between two strains to systematically identify transcription factors whose activity profiles are affected by *fkh1 fkh2 *mutation. This new deviation coefficient is defined as,



where superscripts *wt *and *mt *represent wild type and *fkh1 fkh2 *mutant, respectively, and the overhead bars represent the vector magnitude of TFA. Small values of the deviation coefficient suggest activities dominated by noise. While large values suggest significant activities and significant differences in TFA profiles between mutant and wild-type.

## Authors' contributions

YLY performed the analysis, evaluated the results, and drafted the manuscript. JS carried out spectral analysis of TFAs. MPB performed the network sensitivity investigation and participated in the design of this study. SJG developed the subnetwork generation algorithm. JCL initiated the project, helped with evaluation of the results and the manuscript, and provided mentorship. All authors read and approved the final manuscript.
